# *Pemt* Deficiency Ameliorates Endoplasmic Reticulum Stress in Diabetic Nephropathy

**DOI:** 10.1371/journal.pone.0092647

**Published:** 2014-03-25

**Authors:** Mayu Watanabe, Atsuko Nakatsuka, Kazutoshi Murakami, Kentaro Inoue, Takahiro Terami, Chigusa Higuchi, Akihiro Katayama, Sanae Teshigawara, Jun Eguchi, Daisuke Ogawa, Eijiro Watanabe, Jun Wada, Hirofumi Makino

**Affiliations:** 1 Department of Medicine and Clinical Science, Okayama University Graduate School of Medicine, Dentistry and Pharmaceutical Sciences, Kita-ku, Okayama, Japan; 2 Department of Diabetic Nephropathy, Okayama University Graduate School of Medicine, Dentistry and Pharmaceutical Sciences, Kita-ku, Okayama, Japan; 3 Department of General Medicine, Okayama University Graduate School of Medicine, Dentistry and Pharmaceutical Sciences, Kita-ku, Okayama, Japan; 4 Dainippon Sumitomo Pharma, Chuo-Ku, Osaka, Japan; Duke University Medical Center, United States of America

## Abstract

Phosphatidylethanolamine N-methyltransferase (*Pemt*) catalyzes the methylation of phosphatidylethanolamine (PE) to phosphatidylcholine (PC) mainly in the liver. Under an obese state, the upregulation of *Pemt* induces endoplasmic reticulum (ER) stress by increasing the PC/PE ratio in the liver. We targeted the *Pemt* gene in mice to explore the therapeutic impact of *Pemt* on the progression of diabetic nephropathy and diabetes, which was induced by the injection of streptozotocin (STZ). Although the blood glucose levels were similar in STZ-induced diabetic *Pemt+/+* and *Pemt−/−*mice, the glomerular hypertrophy and albuminuria in *Pemt−/−* mice were significantly reduced. *Pemt* deficiency reduced the intraglomerular F4/80-positive macrophages, hydroethidine fluorescence, tubulointerstitial fibrosis and tubular atrophy. The expression of glucose-regulated protein-78 (GRP78) was enriched in the renal tubular cells in STZ-induced diabetic mice, and this was ameliorated by *Pemt* deficiency. In mProx24 renal proximal tubular cells, the treatment with ER-stress inducers, tunicamycin and thapsigargin, increased the expression of GRP78, which was reduced by transfection of a shRNA lentivirus for *Pemt* (shRNA-Pemt). The number of apoptotic cells in the renal tubules was significantly reduced in *Pemt−/−* diabetic mice, and shRNA-*Pemt* upregulated the phosphorylation of Akt and decreased the cleavage of caspase 3 and 7 in mProx24 cells. Taken together, these findings indicate that the inhibition of *Pemt* activity ameliorates the ER stress associated with diabetic nephropathy in a model of type 1 diabetes and corrects the functions of the three major pathways downstream of ER stress, *i.e.* oxidative stress, inflammation and apoptosis.

## Introduction

Phosphatidylethanolamine N-methyltransferase (*Pemt*) catalyzes the methylation of phosphatidylethanolamine (PE) to phosphatidylcholine (PC) as well as the conversion of S-adenosylmethionine (SAM) to S-adenosylhomocysteine (SAH) [1]. The Pemt protein is localized in the endoplasmic reticulum (ER)-associated membranes in the liver. PC is mainly produced via the CDP-choline pathway, and is synthesized from diet-derived choline in liver tissue, while methionine is converted to SAM, which is the methyl donor for the conversion of PE catalyzed by Pemt. Pemt is responsible for the *de novo* synthesis of PC, accounting for ∼30% of the PC in rodents [2]. The secretion of very low density lipoprotein (VLDL) from hepatocytes is facilitated by the enrichment of PC, and both CDP-choline and the *Pemt* pathways are required for the optimal secretion of VLDL from the liver [3]. Thus, *Pemt−/−* mice demonstrated decreased VLDL secretion, and *Pemt* deficiency strikingly protected *Ldlr−/−* mice from the development of atherosclerosis [4].

Recently, Pemt has been shown to play a critical role in obesity and insulin resistance using *Pemt−/−* mice fed a high-fat diet. The *Pemt−/−* mice were protected from high-fat diet-induced weight gain and insulin resistance over the 10-week experimental period [5] and the deficiency in choline biosynthesis seems to provide the beneficial effects for glucose metabolism. Furthermore, the lipidomic analyses of the ER of the liver in obese mice revealed an increased PC/PE ratio, which was closely associated with inhibition of the sarco/endoplasmic reticulum calcium ATPase (SERCA) activities and the induction of ER stress in mice. The reduction of *Pemt* expression by short hairpin RNA (shRNA) led to the reduction of the PC/PE ratio and beneficial effects on ER stress and improved the glucose metabolism and fatty liver in mice [6].

The increased expression of *Pemt* in the liver under the obese state is linked to the overproduction of SAH, which is metabolized to homocysteine, thus resulting in increased serum levels of homocysteine. A higher serum homocysteine level is a well-known risk factor for cardiovascular disease [7], [8] and progressive kidney disease [9], [10]. Increased homocysteine levels are reported to cause both ER stress and local oxidative stress, which subsequently leads to the induction of glomerular cell dysfunction and glomerulosclerosis [11]. Although decrease the homocysteine level with folic acid and B vitamins did not reduce the risk for major cardiovascular events in patients with vascular diseases [8] or acute myocardial infarction [7], the inhibition of intrinsic *Pemt* activity may be beneficial by directly ameliorating the ER stress and oxidative stress, in addition to the homocysteine lowering effects.

The *Pemt* mRNA and activity are predominantly expressed in the liver. However, low activity of *Pemt* has also been demonstrated in other tissues, such as the heart, kidneys and adipose tissues [1]. In diabetic nephropathy, proteinuria and hyperglycemia induce ER stress [12], [13] and lead to subsequent oxidative stress [14], inflammatory responses [15] and apoptosis [16], [17] in renal tubular cells, which ultimately progress to the end-stage renal disease associated with tubulointerstitial fibrosis. Since the *Pemt* expression has been shown to increase in streptozotocin (STZ)-induced diabetic rats [18], we hypothesized that a deficiency of *Pemt* may protect against the renal injuries associated with diabetic nephropathy by reducing the serum homocysteine levels or by directly ameliorating the ER stress in the kidney. In the present study, we generated *Pemt* knockout mice and demonstrated that the deficiency of *Pemt* protects against diabetic nephropathy by ameliorating the ER stress and subsequent pathways, such as those involving oxidative stress, inflammation and apoptosis.

## Materials and Methods

### Generation of Pemt (phosphatidylethanolamine N-methyltransferase) Knockout Mice

The *Pemt* targeting vector was designed to replace exon 2 of *Pemt* with a PGK-neomycin resistance cassette. The 2.9 kb short (12081–15030) and 6.2 kb long arm (15440–21643) genomic DNA fragments (**Figure S1 in File S1**) were obtained from the C57BL/6 mouse Bac genomic clones (ID: RP23-101K22 and RP23-151K5, Roswell Park Cancer Institute) or the C57BL/6-derived ES genome by PCR using primers containing additional restriction enzyme recognition sites for subcloning; *SacII* and *NotI* for the short arm and *ClaI* and *SalI* for the long arm. The *SacII-NotI-*digested short arm PCR fragment was subcloned into the pBS-pA-loxp-NEO-SH vector (Unitech, Chiba, Japan). The targeting vector was constructed by subcloning the *SacII-ClaI-*digested short arm flanked by SV40 poly A-loxp-NEO-loxp, and the *ClaI-SalI-*digested long arm was subcloned into the pBS-NEO-DTA vector (Unitech, Chiba, Japan) constructed from pBlueScriptII SK+ (Stratagene, La Jolla, CA). The targeting plasmid was linearized by *SacII* digestion, and was electroporated into C57BL/6-derived embryonic stem (ES) cells. The homologous recombination of C57BL/6 ES cell clones was screened by a Southern blot analysis using a 5′-probe, NEO-probe and 3′-probe and *EcoRI*-digested genomic DNA. Chimeric mice were generated by injecting the targeted ES clone into C57BL/6 blastocysts. Male chimeric mice were mated with C57BL/6JJcl mice to generate heterozygous *Pemt+/−* mice, which were backcrossed to C57BL/6JJcl mice for more than five generations. The genotyping of *Pemt−/−* mice was performed by PCR using 5′-ATGAGACTTAAGTGCAGTGACTGTG-3′ and 5′-CTTCCTCGTGCTTTACGGTATC-3′ as the primers, and *Pemt+/+* mice were identified by the 5′-ATGAGACTTAAGTGCAGTGACTGTG-3′ and 5′- GGTACTCACCACATTCCAGAAGAGT -3′ sequences.

### Animals

Male *Pemt−/−* and *Pemt+/+* mice were housed in cages and maintained on a 12-hour light-dark cycle and received normal chow (MF, Oriental Yeast, Co., Ltd). Eight-week-old mice received five consecutive intravenous injections of 50 mg/kg streptozotocin (STZ) (Sigma, St. Louis, MO) in citrate buffer at pH 4.6 or citrate buffer only (as a control). A blood glucose level over 300 mg/dl was confirmed three days after the STZ administration at two different time points. We sacrificed the mice at 25 weeks of age and subjected them to the subsequent studies. All animal experiments were approved by the Animal Care and Use Committee of the Department of Animal Resources, Advanced Science Research Center, Okayama University.

The serum total homocysteine levels were measured by an enzymatic colorimetric assay (Alfresa Co., Tokyo), the quantification of PC and PE was performed by thin-layer chromatography (TORAY Research Center, Tokyo) and the urinary albumin levels were measured using goat-antiserum against mouse albumin (Cappel) and a turbidimetric immunoassay in the BN II System (Siemens, Eschborn, Germany) and were normalized against the creatinine levels.

### Poly (A^+^) RNA Analysis

For the quantitative real time PCR analysis, cDNAs synthesized from 2 μg of total RNA were amplified in the presence of primers and TaqMan Minor Groove Binder Probes (TaqMan Gene Expression Assays; Applied Biosystems, Carlsbad, CA) using a StepOnePlus Real Time PCR System. The relative abundance of *Pemt* mRNA (Mm00839436_m1, Applied Biosystems) was standardized using β-actin mRNA (Mm00607939_s1) as the internal control.

### Cell Culture

Mouse proximal tubular cells (mProx24) were cultured in DMEM with 5% FCS and 100 U/ml penicillin, 100 μg/ml streptomycin and 2 μM L-glutamine [19]. For the knockdown experiments, the mProx24 cells were transfected with 5 MOI (multiplicity of infection) of MISSION shRNA lentivirus transduction particles for *Pemt* (NM_008819) (shRNA-Pemt) or Non-Target shRNA control lentivirus transduction particles (shRNA-CON). Each group of shRNA-Pemt and shRNA-CON was divided into six sub-groups; normal glucose (NG), high glucose (HG), osmotic control for mannitol (Mn), DMSO, tunicamycin-treated and thapsigargin-treated groups. Tunicamycin and thapsigargin were purchased from SIGMA, and the stock solution was prepared in DMSO for the induction of ER stress. Prior to the high glucose stimulus and induction of ER stress, the cells were serum-starved in DMEM with 1% FCS for 24 hours, then the culture media were changed to DMEM containing 1% FCS with NG (5.5 mM glucose), HG (25 mM glucose), Mn (5.5 mM glucose and 19.5 mM mannitol), 1 μg/ml tunicamycin, 1 μM thapsigargin and 0.1% DMSO. After a 24-hour incubation, they were subjected to a Western blot analysis and CellTiter 96 Aqueous One Solution Cell Proliferation Assay (Promega, Madison, WI). All culture experiments were performed in triplicate.

### Western Blot Analyses

Kidney cortex tissues were excised and homogenized with lysis buffer (20 mM Tris-HCl, pH 7.4, 100 mM NaCl, 10 mM benzamidine-HCL, 10 mM ε-amino-*n*-caproic acid, 2 mM phenylmethylsulfonyl fluoride and 1% Triton X-100). After centrifugation at 14,000 rpm for 30 min at 4°C, supernatants were collected for the further analyses. Equal amounts of protein were subjected to SDS-PAGE under reducing conditions, and were electroblotted onto Hybond P polyvinylidine fluoride membranes (GE Healthcare Life Sciences, Pittsburg, PA). The membrane blots were immersed in a blocking solution containing 5% nonfat dry milk and Tris-buffered saline with Tween-20 (0.05% Tween-20, 20 mM Tris-HCl, and 150 mM NaCl, pH 7.6). Then, the membranes were incubated with the following primary antibodies: rabbit polyclonal anti-phospho-elF2α (Ser51), rabbit polyclonal anti-eIF2α, rabbit monoclonal anti-IRE1α (14C10), rabbit monoclonal anti-GAPDH (14C10), anti-phospho-Akt (Ser473), Akt, Caspase-3, Caspase-7 (Cell Signaling Technology, Beverly, MA), rabbit polyclonal to IRE1 (phospho S724), ATF6, XBP-1 (abcam, Cambridge, MA), goat polyclonal anti-GRP78 (C-20), rabbit polyclonal anti-p21(H-164), p27(C-19), cyclin D1(M-20) (Santa Cruz Biotechnology, Dallas, TX) and anti-PEMT antibody (abcam; ab172388). They were then incubated with anti-rabbit or anti-goat IgG conjugated with horseradish peroxidase (GE Healthcare Life Sciences). The blots were washed three times with Tris-buffered saline with Tween-20, immersed in ECL Plus Western Blotting Detection Reagents (GE Healthcare Life Sciences), and then the chemiluminescence was analyzed using the LAS-3000 mini instrument (FUJIFILM, Tokyo, Japan).

### Morphological Studies

Renal tissue specimens were fixed in 10% formaldehyde and embedded in paraffin, and 4 μm-thick sections were prepared. The sections were stained with periodic acid-Schiff (PAS) and Masson-Trichrome. To evaluate the glomerular size, we examined 15 randomly selected glomeruli per animal at 16 weeks after the induction of diabetes. The area of the glomerular tuft and the mesangial matrix index (MMI) were measured using the Lumina Vision software program (Mitani Corporation, Tokyo, Japan). The MMI was defined as the PAS-positive area in the tuft area, calculated using the following formula: MMI = (PAS positive area)/(tuft area). The results are expressed as the means ± SE. Immunofluorescence staining was performed using Collagen Type IV (C-19) and TGF-β1/2/3 (H-112) (Santa Cruz Biotechnology), and the type IV collagen expression was quantified using the Lumina Vision software program. Four μm-thick sections of formalin-fixed, paraffin-embedded tissues were deparaffinized and rehydrated, and sections were pretreated by microwaving them for 10 minutes in Target Retrieval Solution (DAKO) for antigen retrieval. Nonspecific binding was blocked by incubation for 30 min in 10% goat or rabbit serum, as appropriate. The tissues were then incubated with a rat monoclonal anti-F4/80 antibody [CI:A3-1] (abcam), anti-PEMT antibody (abcam; ab172388) or BiP (C50B12) rabbit monoclonal antibody (Cell signaling Technologies) at 4°C overnight. After being washed in PBS, the sections were incubated with a biotinylated secondary antibody, the VECTASTAIN ABC Standard Kit (Vector Laboratories, Burlingame, CA). Immunochemical staining was performed with the ImmPACT DAB SUBSTRATE (Vector Laboratories, Burlingame, CA).

### Oxidant Fluorescence Microtopography

The intracellular generation of O^2−^ was assessed using hydroethidine. O^2−^ reacts with hydroethidine to produce ethidium bromide, which binds to nuclear DNA and gives red fluorescence. Unfixed frozen kidneys were cut into 30 μm-thick sections and placed on a glass slide. Hydroethidine (2×10^−6^ M in PBS) was applied to each tissue section. Slides were incubated in a light-protected and humidified chamber at 37°C for 30 minutes. Images were obtained with a fluorescence microscope (BZ-8100, KEYENCE, Osaka). All tissue specimens were processed and imaged in parallel.

### Statistical Analysis

The data are expressed as the means ± s.e.m., and the multiple comparisons were performed by a one-way ANOVA with Bonferroni and Tukey corrections. A value of *P*<0.05 was regarded as statistically significant. The data were analyzed using the IBM SPSS Statistics software program (IBM, Armonk, NY).

## Results

### Pemt Deficiency Reduces Albuminuria in STZ-induced Diabetic Mice

**Figure 1:**
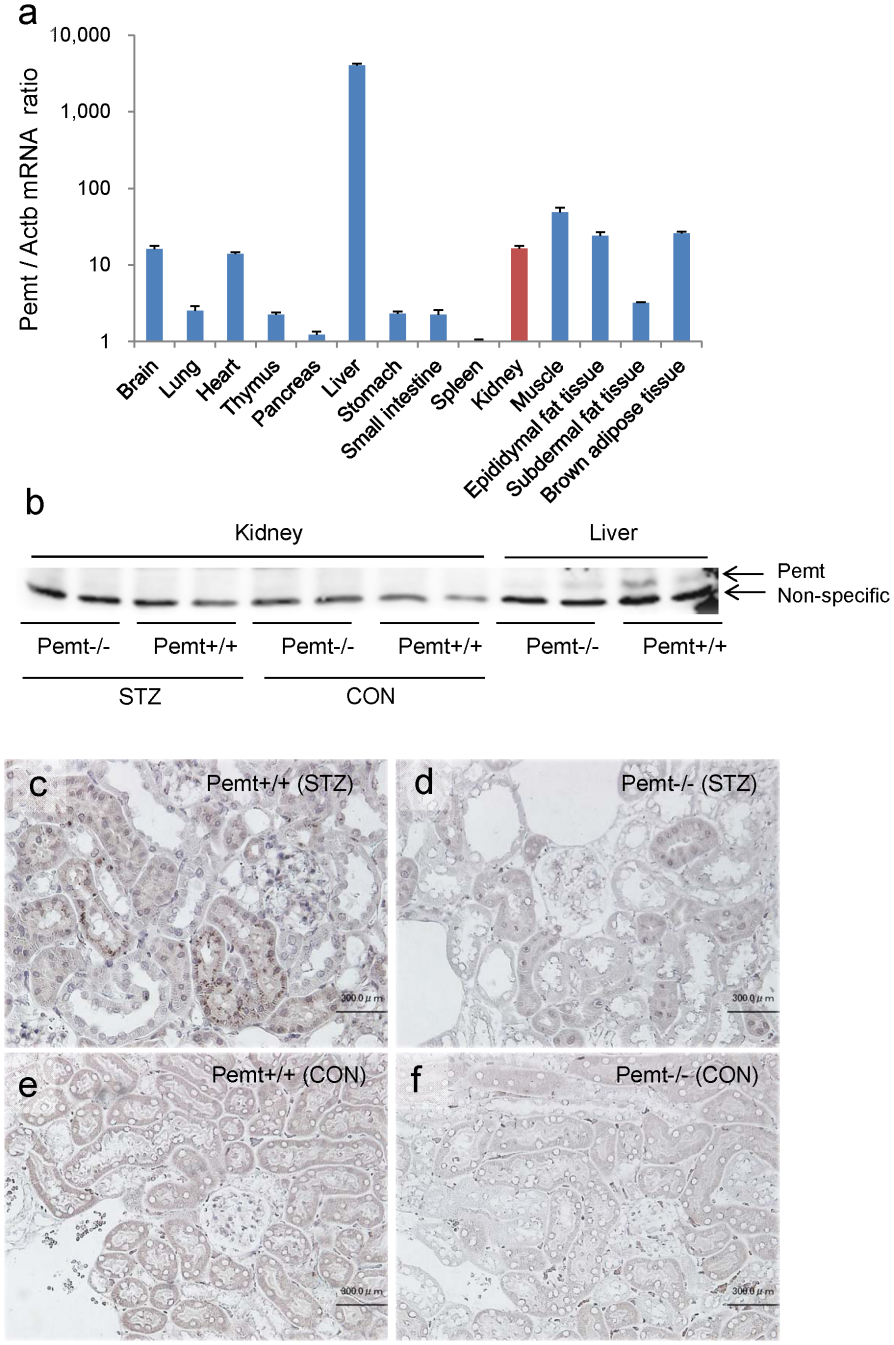
The mRNA and protein expression of phosphatidylethanolamine N-methyltransferase (*Pemt*). **a.** The results of the quantitative RT-PCR assay for *Pemt* mRNA in various tissues of C57BL/6JJcl mice (n = 4). **b.** The Western blot analysis of Pemt in the kidney and liver tissues of *Pemt+/+* mice and *Pemt−/−* C57BL/6JJcl mice. **c–f.** Immunoperoxidase staining for Pemt in the kidney tissue of *Pemt+/+* mice and *Pemt−/−* mice. Immunoreactivity was seen in the proximal tubules and glomerular cells in streptozotocin (STZ)-treated *Pemt+/+* mice (**c**), but not in control *Pemt+/+* mice (**e**) or *Pemt−/−* mice (**d and f**). Bars = 50 μm **(c–f**).

To explore the role of *Pemt* in the progression of diabetic nephropathy, *Pemt−/−* mice were generated by a standard gene targeting method (**Figure in File S1**). The *Pemt−/−* mice did not exhibit any abnormalities in their general appearance, and all animals fed normal chow were healthy until 48 weeks of age. In the *Pemt+/+* mice, *Pemt* mRNA was predominantly expressed in the liver, but was also detected in the kidneys, brain, heart, skeletal muscle and adipose tissues (**Figure 1a**). Pemt protein was detected in the liver tissues, and was barely detected in the kidney tissues of *Pemt+/+* mice by a Western blot analysis (**Figure 1b**). However, in the histochemical examinations, immunoreactivity for *Pemt* was demonstrated mainly in the cytoplasm of the tubular cells and also in the glomerular cells in STZ-induced diabetic *Pemt+/+* mice (**Figure 1c**), while no such staining was detected in control *Pemt+/+* and *Pemt−/−* mice (**Figures 1d–f**).

**Figure 2:**
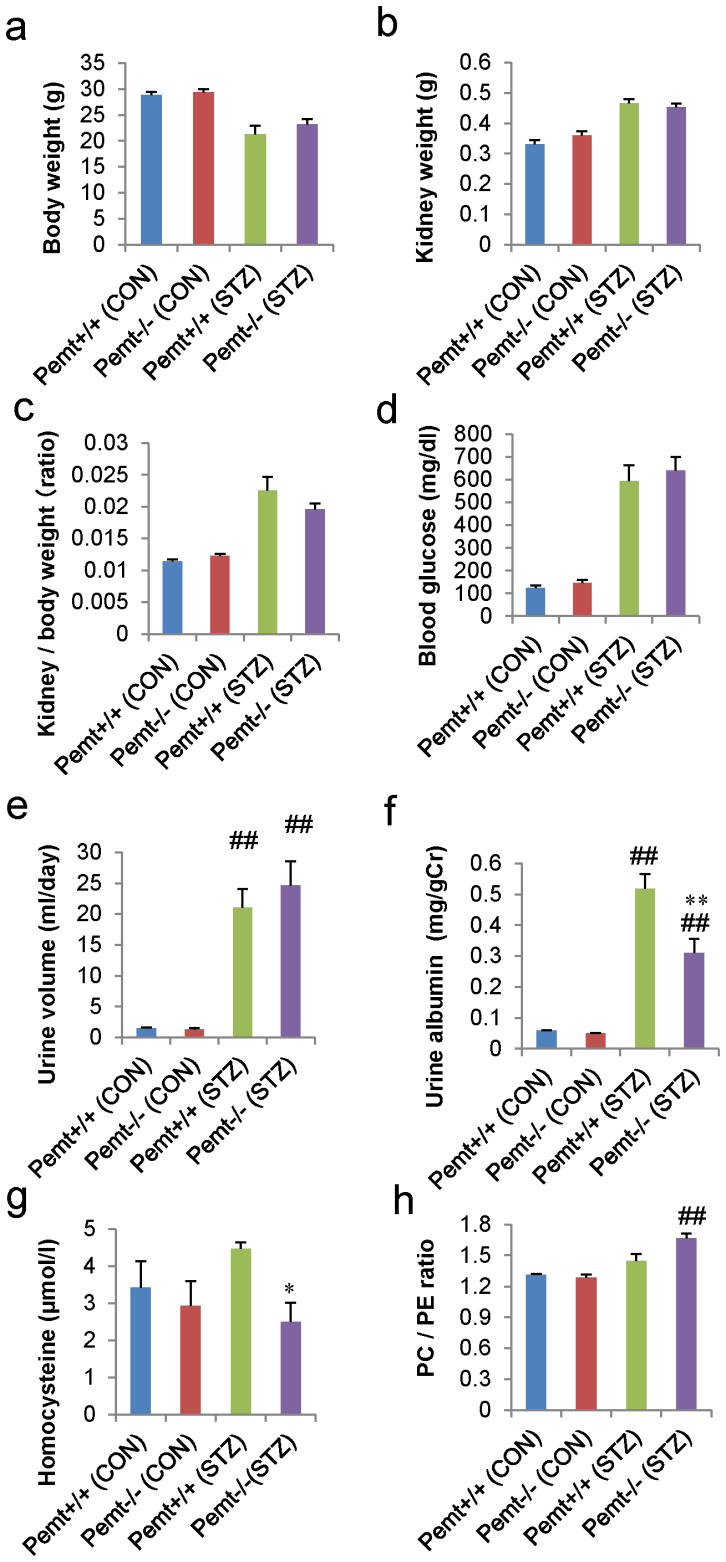
The metabolic data and findings of albuminuria in streptozotocin (STZ)-treated diabetic *Pemt+/+* and *Pemt−/−* C57BL/6JJcl mice. The *Pemt+/+* and *Pemt−/−* mice were treated with citrate buffer (CON) or streptozotocin (STZ). **a.** Body weight (g), **b.** kidney weight (g), **c.** kidney/body weight, **d.** blood glucose levels (mg/dl), **e.** urine volume (ml/day), **f.** albumin level (mg/gCr) and **g.** serum homocysteine level (μmol/l). The daily albumin excretion and serum homocysteine levels were significantly reduced in *Pemt−/−* (STZ) mice compared with *Pemt+/+* (STZ) mice. **h.** The phosphatidylethanolamine (PE) and phosphatidylcholine (PC) contents were measured in the kidney tissues by thin-layer chromatography. The PC/PE ratio was significantly increased in *Pemt−/−* (STZ) compared with *Pemt−/−* (CON) mice; however, there was no statistically significant difference between the *Pemt−/−* (STZ) and *Pemt+/+* (STZ) mice. ^##^P<0.01 v.s. *Pemt+/+* (CON). **P<0.01, *P<0.05 v.s. *Pemt+/+* (STZ).

After the induction of diabetes by STZ, mice were sacrificed at 25 weeks of age. The blood glucose levels were similar in both STZ-induced diabetic *Pemt+/+* and *Pemt−/−* mice. The kidney/body weight ratios of the diabetic *Pemt−/−* mice tended to be reduced compared with those of Pemt+/+ mice; however, the difference did not reach statistical significance (**Figures 2a–d**). Osmotic diuresis was observed in both *Pemt+/+* and *Pemt−/−* diabetic mice, but the albuminuria was significantly suppressed in diabetic *Pemt−/−* mice compared with *Pemt+/+* mice (0.31±0.05 *v.s.* 0.52±0.06 mg/gCr, p = 0.007) (**Figures 2e and f**).

*Pemt* converts S-adenosylmethionine (SAM) to S-adenosylhomocysteine (SAH), and SAH is metabolized to homocysteine, which is coupled with the conversion from PE to PC. The measurement of the serum homocysteine levels demonstrated that they were significantly reduced in diabetic *Pemt−/−* mice compared with *Pemt+/+* mice (2.50±0.52 *v.s.* 4.47±0.18 μmol/l, p = 0.035) (**Figure 2g**). In contrast, the PC/PE ratio was increased in *Pemt−/−* mice treated with STZ, and the *Pemt* deficiency did not reduce the PC/PE ratio in the diabetic *Pemt−/−* mice (**Figure 2h**).

### Pemt Deficiency Ameliorates Glomerular Hypertrophy, Oxidative Stress, Inflammation and the Accumulation of Extracellular Matrix in STZ-induced Diabetic Mice

**Figure 3:**
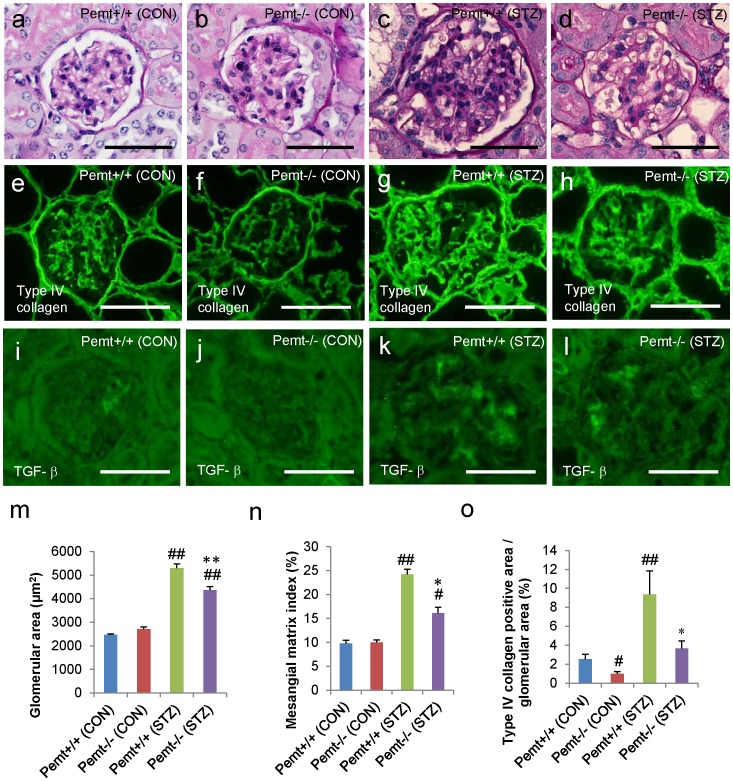
The results of the histochemical and morphometric analyses of streptozotocin (STZ)-treated diabetic *Pemt+/+* and *Pemt−/−* C57BL/6JJcl mice. The *Pemt+/+* and *Pemt−/−* mice were treated with citrate buffer (CON) or streptozotocin (STZ). **a–d.** Periodic acid-Schiff stain, **e–h.** immunofluorescence staining for type IV collagen, **i–l.** immunofluorescence staining for TGF-β, **m.** the glomerular area (μm^2^), **n.** the mesangial matrix index (%) and **o.** the type IV collagen positive area/glomerular area (%). The glomerular hypertrophy, mesangial area and type IV collagen positive area were significantly reduced in *Pemt−/−* (STZ) mice compared with *Pemt+/+* (STZ) mice. Bars = 50 μm (**a–l**). ^##^P<0.01, ^#^P<0.05 v.s. *Pemt+/+* (CON). **P<0.01, *P<0.05 v.s. *Pemt+/+* (STZ).

The glomerular hypertrophy was significantly reduced in STZ-induced diabetic *Pemt−/−* mice compared with *Pemt+/+* mice (4367±141 *v.s.* 5293±186 μm^2^, p = 0.003) (**Figures 3a–d and 3m**). The mesangial matrix index and accumulation of intraglomerular type IV collagen was also significantly reduced in STZ-induced diabetic *Pemt−/−* mice compared with *Pemt+/+* mice (**Figures 3e, f, n and o**). The kidneys from the STZ-treated *Pemt+/+* mice demonstrated prominent increases in hydroethidine fluorescence compared with those from control *Pemt+/+* mice; *Pemt* deficiency markedly reduced the renal cell-derived superoxide (**Figure in File S1**). The immunoreactivity for transforming growth factor-β (TGF-β) in the glomeruli was also reduced in the diabetic *Pemt−/−* mice (**Figures 3i–l**).

**Figure 4:**
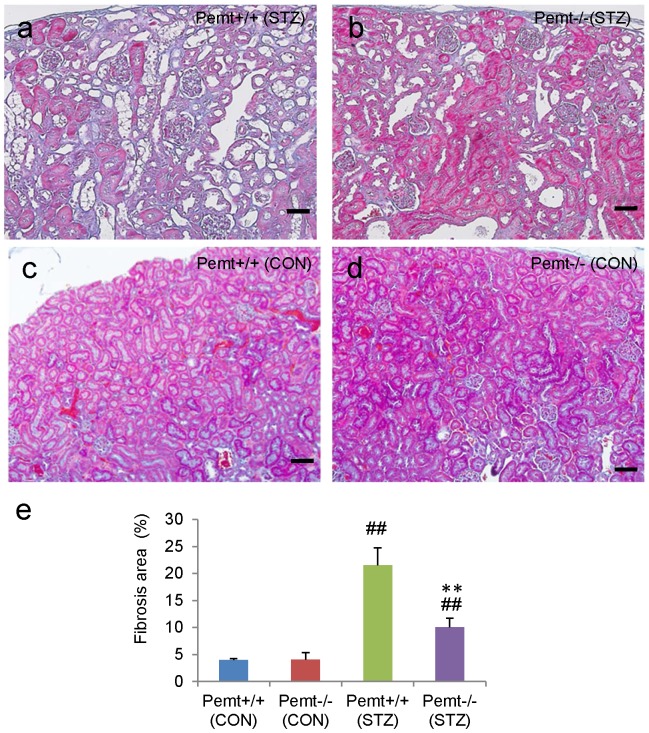
The tubulointerstitial injuries in streptozotocin (STZ)-treated diabetic *Pemt+/+* and *Pemt−/−* C57BL/6JJcl mice. The *Pemt+/+* and *Pemt−/−* mice were treated with citrate buffer (CON) or streptozotocin (STZ). **a–d.** Periodic acid-Schiff stain. The tubular atrophy, dilatation and interstitial fibrosis were ameliorated in *Pemt−/−* (STZ) mice compared with *Pemt+/+* (STZ) mice. Bars = 300 μm **(a–d**). e. Fibrosis area (%).^##^P<0.01 v.s. *Pemt+/+* (CON). **P<0.01 v.s. *Pemt+/+* (STZ).

We further investigated the accumulation of F4/80 cells in the glomeruli, and the interstitial macrophage infiltration was significantly reduced in STZ-induced diabetic *Pemt−/−* mice compared with *Pemt+/+* mice (**Figures S3 and S4 in File S1**). In addition to the amelioration of the pathological changes of glomeruli, *Pemt* deficiency also reduced the interstitial pathological alterations such as tubular atrophy, tubular dilatation and interstitial fibrosis (**Figure 4**).

### Pemt Deficiency Ameliorates Endoplasmic Reticulum Stress in Diabetic Nephropathy

**Figure 5:**
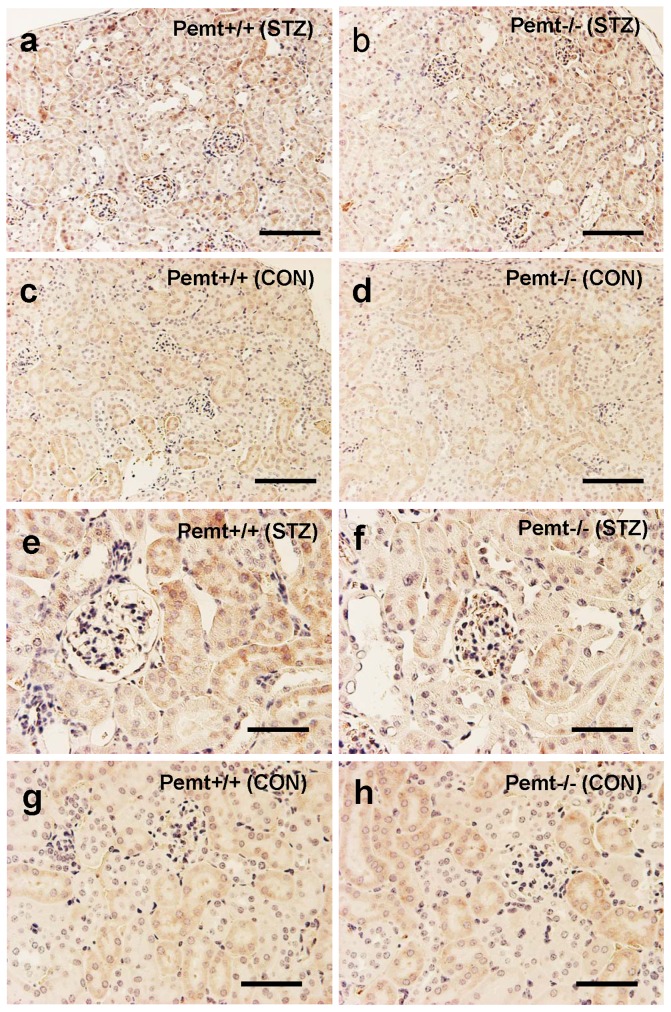
The expression of 78-regulated protein (GRP78) in streptozotocin (STZ)-treated diabetic *Pemt+/+* and *Pemt−/−* C57BL/6JJcl mice. The *Pemt+/+* and *Pemt−/−* mice were treated with citrate buffer (CON) or streptozotocin (STZ). **a–d.** Immunoperoxidase staining for GRP78 (Bars = 300 μm), **e–h.** Immunoperoxidase staining for GRP78 (Bars = 100 μm). The immunoreactivity for GRP78 was widely distributed in the cytoplasm of tubular cells in both non-diabetic *Pemt+/+* mice and *Pemt−/−* mice, and was upregulated in STZ-treated *Pemt+/+* mice.

*Pemt* catalyzes the conversion of phosphathidylethanolamine to phosphatidylcholine, which is coupled with the conversion of SAM to SAH. It has been reported that diet-induced obesity increased the expression of *Pemt* and the PC/PE ratio in the liver, which impaired the ER homeostasis and induced ER stress. We therefore further evaluated the ER stress markers in the kidney tissues and cultured mouse proximal tubular cells (mProx24). We first investigated the localization and expression of a representative ER stress marker, 78 kDa glucose-regulated protein (GRP78). The immunoreactivity for GRP78 was widely distributed in the cytoplasm of tubular cells in both non-diabetic *Pemt+/+* mice and *Pemt−/−* mice, and it was upregulated in STZ-treated *Pemt+/+* mice (**Figures 5a, c and d**). *Pemt* deficiency suppressed the upregulation of GRP78 in the tubular cells (**Figure 5b**). In glomerular cells, the expression of GRP78 was also enhanced by the induction of diabetes in both *Pemt+/+* and *Pemt−/−* mice (**Figures 5e–h**). Although the size of the glomeruli was small, the expression of GRP78 was readily recognized in *Pemt−/−* mice, and there was no clear downregulation of GRP78 in the glomeruli.

**Figure 6:**
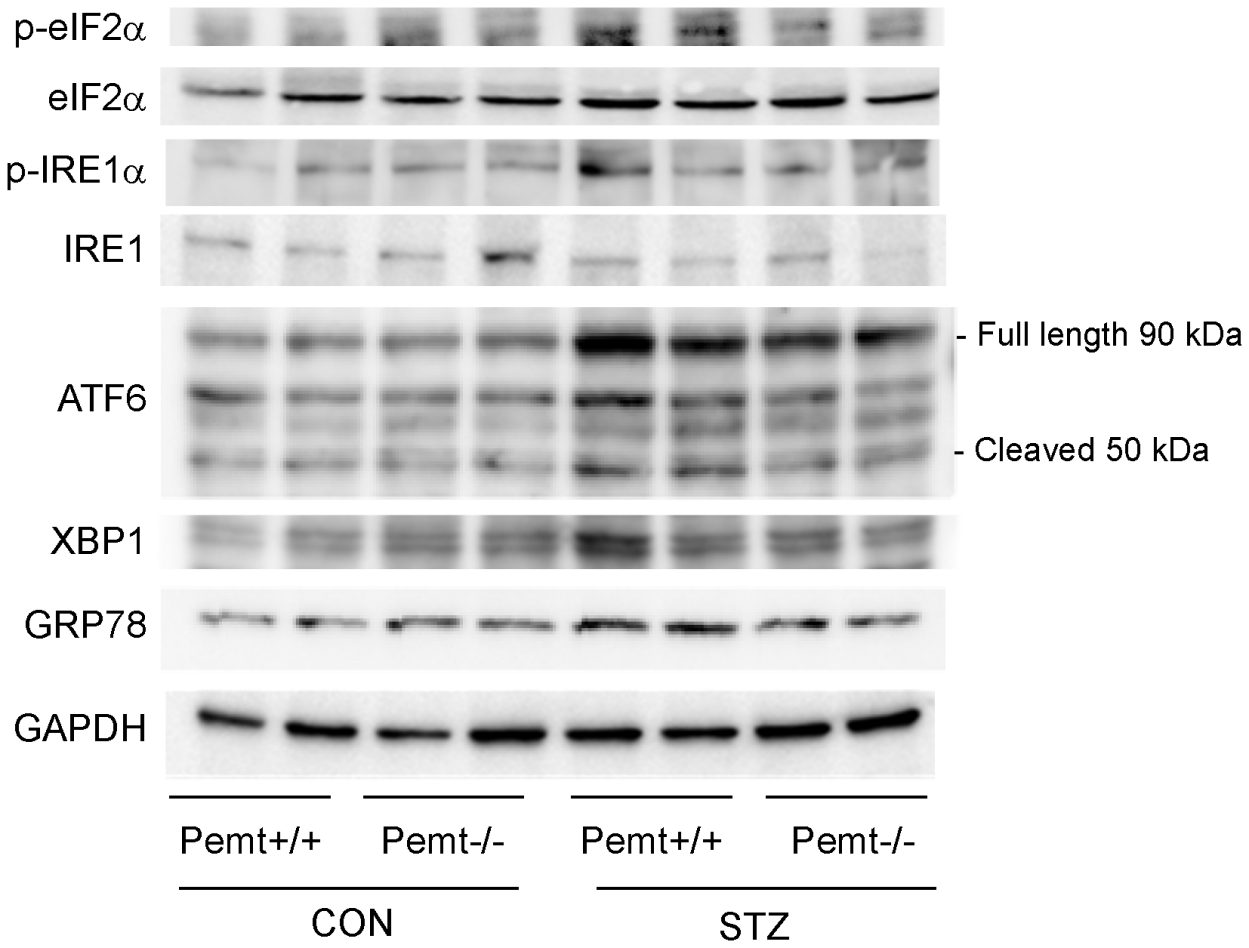
The results of the Western blot analyses of the renal cortex tissues from *Pemt+/+* and *Pemt−/−* mice treated with citrate buffer (CON) or streptozotocin (STZ). The upregulation of ATF6 and GRP78 in *Pemt+/+* diabetic mice was reduced in *Pemt−/−* diabetic mice. The induction of p-eIF2α, p-IRE1α and XBP-1 by STZ treatment was ameliorated by the *Pemt* deficiency; however the difference between the groups did not reach statistical significance (**Supplemental** **Figure 5**).

To quantify the levels of ER-stress markers, we next performed a Western blot analysis. The upregulation of ATF6 and GRP78 in *Pemt+/+* diabetic mice was significantly reduced in *Pemt−/−* diabetic mice (**Figure 6** **and** **Figure S5 in File S1**). The induction of p-eIF2α, p-IRE1α and XBP-1 by STZ treatment was ameliorated by the *Pemt* deficiency; however, the difference did not reach statistical significance (**Figure 6** **and** **Figure S5 in File S1**).

We next investigated cultured mProx24 cells treated with shRNA-CON (control) and shRNA-Pemt (**Figure S6 in File S1**). In the cultured mProx24 renal tubule cells, the treatment with a high glucose level did not alter the expression of *Pemt* or various ER stress markers, such as IRE1α, eIF2α, ATF6, XBP-1 and GRP78. In contrast, treatment with tunicamycin and thapsigargin enhanced the expression of GRP78 and the phosphorylation of IRE1α and elF2α. The treatment with shRNA-Pemt led to a ∼30% reduction of Pemt, and it significantly suppressed the upregulation of GRP78 induced by tunicamycin and thapsigargin treatment. However, shRNA-Pemt did not suppress the ER stress-induced phosphorylation of IRE1α and elF2α (**Figure S6 in File S1**).

### Pemt Deficiency Ameliorates Apoptosis under Endoplasmic Reticulum (ER) Stress

*Pemt* is known to downregulate the PI3K/Akt pathway and induce apoptosis in liver cells [20]. In addition, hyperglycemia and oxidative stress upregulate the PI3K/Akt pathway, which is associated with early phase hyperplasia and apoptosis in the proximal tubular cells [21]. We next investigated the status of cell proliferation and apoptosis of tubular cells under ER stress. In the cultured mProx24 cells, the treatment with high glucose media did not alter the expression of cyclin D1, p21^Cip1^, p27^Kip1^ or p-Akt (**Figures S7 and S8 in File S1**). In contrast, the treatment with tunicamycin and thapsigargin upregulated the expression of cyclinD1, downregulated p27^Kip1^ and p-Akt and inhibited the cell proliferation. Under ER stress induced by tunicamycin or thapsigargin, the treatment with shRNA-Pemt upregulated the level of p-Akt; however, it did not alter the proliferation of mProx24 cells.

**Figure 7:**
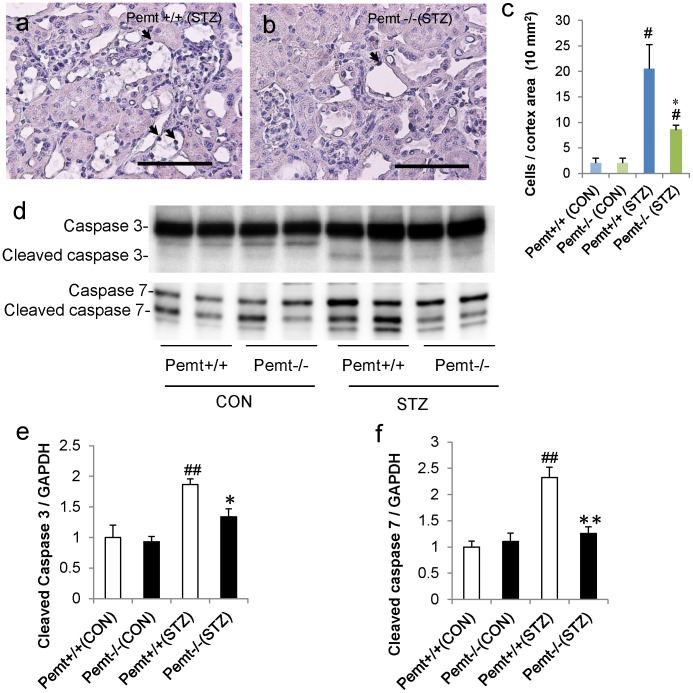
Apoptosis in the streptozotocin (STZ)-treated diabetic *Pemt+/+* and *Pemt−/−* C57BL/6JJcl mice and in mProx24 cells treated with MISSION shRNA lentivirus transduction particles for *Pemt* (shRNA-Pemt) and Non-Target shRNA control lentivirus transduction particles (shRNA-CON). **a.** and **b.** The results of the *in situ* TUNEL assay in *Pemt−/−* (STZ) and *Pemt+/+* (STZ) mice. Bars = 100 μm. **c.** The quantification of apoptotic cells in the kidney cortex. The number of TUNEL-positive cells was significantly reduced in the *Pemt−/−* (STZ) mice compared with *Pemt+/+* (STZ) mice. ^#^P<0.05; STZ-treated group (STZ) *v.s.* citrate buffer treated control group (CON). *P<0.05; *Pemt+/+* (STZ) v.s. *Pemt−/−* (STZ). **d.** The results of the Western blot analyses of caspases 3 and 7 in the renal cortex tissues of *Pemt+/+* and *Pemt−/−* mice. **e.** The increase in cleaved caspases 3 and 7 was ameliorated by the deficiency of *Pemt* in STZ-treated mice. ^##^P<0.01, ^#^P<0.05 v.s. *Pemt+/+* (CON). **P<0.01, *P<0.05 v.s. *Pemt+/+* (STZ).

We next evaluated the status of apoptosis in tubular cells. In the diabetic kidney, the number of TUNEL-positive apoptotic tubular cells was significantly reduced in *Pemt−/−* mice compared with *Pemt+/+* mice (**Figure 7**). In cultured mProx24 cells, the treatment with tunicamycin or thapsigargin increased the levels of cleaved caspases 3 and 7, and shRNA-Pemt also reduced the cleavage of caspases 3 and 7 compared with shRNA-CON (**Figure S9 in File S1**).

## Discussion

ER stress is induced by the accumulation of *de novo* synthesized unfolded proteins, which activate the unfolded protein response (UPR). The three major arms of the UPR include the PKR-like eukaryotic initiation factor 2α kinase (PERK), inositol requiring enzyme 1 (IRE1α) and activating transcription factor-6 (ATF6) pathways. Upon accumulation of misfolded proteins in the ER or depletion of ER calcium stores, ATF6 is released from GRP78 and cleaved by site-1 and site-2 proteases. Then, the fragments migrate to the nucleus and activate ER chaperones and enzymes that promote protein folding and the ER-associated degradation (ERAD). IRE1α becomes autophosphorylated and splices X-box binding protein 1 (XBP1) mRNA to yield a potent transcriptional activator. During the UPR, PERK phosphorylates eIF2α and the process reduces the initiation AUG codon recognition and the general rate of translation is reduced [22]. Although the induction of the UPR allows cells to recover from stress, prolonged ER stress may be cytotoxic and lead to apoptosis [23]. The major proapoptotic effector molecules associated with prolonger ER stress are ATF4-mediated induction of C/EBP homologous protein-10 (CHOP/GADD153) and c-Jun N-terminal kinase (JNK) [23]. ATF4 and JNK are preferentially transcribed by the activation of eIF2α and IRE1α, respectively [12]. In addition, the canonical UPR is linked to additional cellular insults, including inflammatory and stress signal systems such as NFκB (nuclear factor κB)-IκB kinase (IKK) and the oxidative stress responses [15].

A number of groups demonstrated upregulation of the ER stress response in diabetic nephropathy in an animal model of diabetes [12]. In STZ-treated rats, increased expression of GRP78 in both tubular and glomerular cells enhanced the expression of CHOP, JNK and caspase-12, and prominent kidney cell apoptosis was demonstrated [24]. In addition, the microarray data from biopsy samples of cases with established diabetic nephropathy revealed higher expression of GRP78, oxygen-regulated protein150 (ORP150/HYOU1) and XBP-1 compared with the cases of mild diabetes [13]. In addition to these observational studies, functional experiments using gene targeting in mice demonstrated the important roles of GRP78 in the progression of renal disease. The knock-in mice that expressed a mutant GRP78, in which the retrieval sequence to the ER was deleted, showed significant tubulointerstitial lesions with aging and chronic protein overload [25]. GRP78 serves as a master regulator of the UPR sensors, ATF6, IRE1α, as well as PERK, and plays an important role in the progression of diabetic nephropathy.

The deficiency of *Pemt* in the liver reversed the increased ER PC/PE ratio, relieved ER stress and improved the systemic glucose homeostasis in a previous study of obese animals [6]. In the liver, the reduction of the PC/PE ratio by *Pemt* deficiency causes the inhibition of SERCA activity, which results in an amelioration of ER stress [6]. However, in the kidney tissues, we did not observe any significant reduction of PC/PE ratio in the *Pemt*−/− mice treated with STZ. Therefore, alternative mechanisms may operate to reduce the ER stress in different tissues. The reduction of the homocysteine levels by *Pemt* deficiency in our investigation may be linked to the amelioration of ER stress, since homocysteine has been reported as an inducer of ER stress through the depletion of Ca^2+^ in the ER [26]. In STZ-induced diabetic mice, the glucose metabolism was not altered by *Pemt* deficiency. However, the ER stress in the kidney tissues was ameliorated and the expression of GRP78 was reduced. In the current investigation, we demonstrated that the amelioration of ER stress by *Pemt* deficiency corrected the three major consequences of ER stress; oxidative stress [14], the inflammatory responses [15] and apoptosis [16], [17].

The alterations in the oxidative environment of the ER and the calcium concentration in the ER cause the production of ER stress-induced reactive oxygen species. For a protein to fold into the correct conformation in the ER, the formation of intramolecular and intermolecular disulfide bonds is required [27]. Electron transport during the disulfide bond formation is driven by a protein relay involving ER-resident enzymes; protein disulfide isomerase (PDI) and ER oxidoreductin 1 (ERO1) [28]. Although it provides a robust driving force for disulfide bond formation, the consumption of oxygen for the terminal electron leads to the production of ROS [15]. Furthermore, the mitochondria contribute to lethal levels of ROS during the sustained ER stress by affecting the mitochondrial electron transfer system [14]. Hyperglycemia also contributes to the ROS production from mitochondria by enhancing the supply and influx of pyruvate and generating high electrochemical potential [29]. The production of ROS is tightly linked to the inflammatory responses, and promotes the activation of caspase-3 and caspase-12, and also induces tubular apoptosis [30].

ER stress induces the activation of transcription factor such as nuclear factor-κB and JNK, and stimulates the inflammatory responses [15]. The amelioration of ER stress by *Pemt* deficiency inhibited the intraglomerular macrophage infiltration, the accumulation of extracellular matrix and subsequent tubulointerstitial fibrosis. Microinflammation and subsequent extracellular matrix expansion are common pathways for the progression of diabetic nephropathy. In recent years, many researchers have demonstrated that the inflammation pathways play central roles in the progression of diabetic nephropathy, and the identification of the inflammatory molecules involved in this process may lead to the development of new therapeutic strategies [31]. The molecules related to the inflammation pathways in diabetic nephropathy include transcription factors, proinflammatory cytokines, chemokines, adhesion molecules, Toll-like receptors, adipokines and nuclear receptors, which are candidate molecular targets for the treatment of diabetic nephropathy [31]. The inhibition of *Pemt* and amelioration of ER stress is an emerging target for treating the microinflammation in diabetic nephropathy.

During the process of ER stress, ROS, caspase-3, caspase-12 [30], and mammalian target of rapamycin (mTOR) [32] promote the induction of apoptosis. In contrast, the downregulation of Akt signaling [21] and knock-out of CHOP in mice [17] were demonstrated to facilitate the process of ER-induced apoptosis. In the case of diabetic nephropathy, long-term hyperglycemia downregulates the Akt activation and contributes to enhanced p38 mitogen-activated protein kinase (MAPK) activation and the apoptosis of renal tubular cells [21]. In our study, we observed that *Pemt* deficiency relieved the ER stress, activated the phosphorylation of Akt and prominently reduced the apoptosis of proximal tubular cells. The relationship between *Pemt* and Akt signaling was already reported in hepatocytes, where the overexpression of *Pemt* downregulated the PI3K/Akt signaling [20].

Taken together, the inhibition of *Pemt* activity appears to ameliorate the ER stress associated with diabetic nephropathy, and to correct the subsequent three major pathways downstream of ER stress, *i.e.* oxidative stress, inflammation and apoptosis. During the search for small molecules that upregulate GRP78 expression, GRP78 inducers such as trans-4,5-dihydroxy-1,2-dithiane (DTTox) and BiP inducer X (BIX) were identified [33]. In addition, extensive efforts were made to identify chemical chaperones. These studies demonstrated that 4-phenylbutyrate (4-PBA) improves the ER folding capacity and facilitates the trafficking of unfolded proteins, and the endogenous bile acid derivatives, such as tauroursodeoxycholic acid (TUDCA), also protect cells against ER stress-induced apoptosis [34]. The current study suggests that the identification of small molecules which inhibit the *Pemt* activity may be useful in the amelioration of ER stress and ER stress-induced apoptosis in diabetic nephropathy. In addition to our findings, *Pemt* inhibition has been previously reported to have therapeutic potential for insulin resistance and obesity, and also for the prevention of atherosclerosis [4], [6]. Therefore, Pemt inhibitors may be useful in the treatment of diabetic nephropathy in patients with type 1 diabetes, as well as for other ER stress- and oxidative stress-related diseases.

## Supporting Information

File S1 Figure S1. A schematic diagram of the strategy used to disrupt the mouse *Pemt* gene. a. Schematic drawings of the *Pemt* targeting vector. b. The targeted recombination in ES cells (lines 135, 144, 152 and 162) was confirmed by the Southern blot analyses of genomic DNA digested with *EcoRI*, and the expected sizes of wild-type and *Pemt* gene-targeted bands using a 5′-probe, 3′-probe and NEO-probe are shown, respectively. Figure S2. Oxidant fluorescence microtopography using hydroethidine in streptozotocin (STZ)-treated diabetic *Pemt+/+* and *Pemt−/−* C57BL/6JJcl mice. The *Pemt+/+* and *Pemt−/−* mice were treated with citrate buffer (CON) or STZ. a–d. The *Pemt+/+* (STZ) mice demonstrated a prominent increase in hydroethidine fluorescence, and *Pemt* deficiency markedly reduced the renal cell-derived superoxide. Bars = 300 μm (a–d). Figure S3. The intraglomerular macrophage infiltration in streptozotocin (STZ)-treated diabetic *Pemt+/+* and *Pemt−/−* C57BL/6JJcl mice. *Pemt+/+* and *Pemt−/−* mice were treated with citrate buffer or STZ. a–d. Immunoperoxidase staining for F4/80, e. The number of F4/80 positive cells/glomerulus. The number of glomerular F4/80-positive cells was significantly reduced in *Pemt−/−* (STZ) mice compared with *Pemt+/+* (STZ) mice. Bars = 20 μm (a–d). ^##^P<0.01 v.s. *Pemt+/+* (CON). **P<0.01 v.s. *Pemt+/+* (STZ). Figure S4. The interstitial macrophage infiltration in streptozotocin (STZ)-treated diabetic *Pemt+/+* and *Pemt−/−* C57BL/6JJcl mice. *Pemt+/+* and *Pemt−/−* mice were treated with citrate buffer or STZ. a–d. Immunoperoxidase staining for F4/80, e. The number of F4/80 positive cells in the interstitium per mm^2^. The number of interstitial F4/80-positive cells was significantly reduced in *Pemt−/−* (STZ) mice compared with *Pemt+/+* (STZ) mice. Bars = 50 μm (a–d). ^##^P<0.01 v.s. *Pemt+/+* (CON). **P<0.01 v.s. *Pemt+/+* (STZ). Figure S5. The results of the densitometric analyses of the Western blots of renal cortex tissues from *Pemt+/+* and *Pemt−/−* mice treated with citrate buffer or streptozotocin (STZ), which appeared in Figure 6. ^##^P<0.01 v.s. *Pemt+/+* (CON). **P<0.01, *P<0.05 v.s. *Pemt+/+* (STZ). Figure S6. Endoplasmic reticulum (ER) stress-related markers in mProx24 cells treated with MISSION shRNA lentivirus transduction particles for *Pemt* (shRNA-Pemt) and Non-Target shRNA control lentivirus transduction particles (shRNA-CON). The mProx24 renal tubule cells were treated with normal glucose (NG), high glucose (HG), an osmotic control using mannitol (Mn), DMSO, tunicamycin or thapsigargin. **P<0.01, *P<0.05 v.s. shRNA-CON. Figure S7. The expression levels of cyclin D1, p21^Cip1^, p27^Kip1^ and p-Akt in mProx24 cells treated with MISSION shRNA lentivirus transduction particles for *Pemt* (shRNA-Pemt) and Non-Target shRNA control lentivirus transduction particles (shRNA-CON). The mProx24 cells were treated with normal glucose (NG), high glucose (HG), an osmotic control using mannitol (Mn), DMSO, tunicamycin or thapsigargin. The treatments with tunicamycin and thapsigargin upregulated cyclin D1 and downregulated p27^Kip1^ and p-Akt. shRNA-Pemt led to prominent recovery and increased the expression of p-Akt. However, the anti-proliferative activities of tunicamycin and thapsigargin were not reversed by the treatment with shRNA-Pemt, as revealed by the CellTiter96 Aqueous One Solution Cell Proliferation Assay. Figure S8. The results of the densitometric analyses of the Western blot analyses of the levels of cyclin D1, p21^Cip1^, p27^Kip1^ and p-Akt in mProx24 cells treated with MISSION shRNA lentivirus transduction particles for *Pemt* (shRNA-Pemt) and Non-Target shRNA control lentivirus transduction particles (shRNA-CON). **P<0.01, *P<0.05 v.s. shRNA-CON. Figure S9. The densitometric analyses of the Western blots of caspases 3 and 7 in mProx24 cells treated with MISSION shRNA lentivirus transduction particles for *Pemt* (shRNA-Pemt) and Non-Target shRNA control lentivirus transduction particles (shRNA-CON). The treatments with tunicamycin and thapsigargin increased the levels of cleaved caspases 3 and 7, while the treatment with shRNA-Pemt reduces the levels of cleaved caspases 3 and 7. **P<0.01, *P<0.05 v.s. shRNA-CON.
(PDF)

## References

[1] Vance DE (2013) Physiological roles of phosphatidylethanolamine N-methyltransferase. Biochim Biophys Acta 1831: 626–632. 22877991 10.1016/j.bbalip.2012.07.017

[2] DeLong CJ, Shen YJ, Thomas MJ, Cui Z (1999) Molecular distinction of phosphatidylcholine synthesis between the CDP-choline pathway and phosphatidylethanolamine methylation pathway. J Biol Chem 274: 29683–29688. 10514439 10.1074/jbc.274.42.29683

[3] Noga AA, Vance DE (2003) A gender-specific role for phosphatidylethanolamine N-methyltransferase-derived phosphatidylcholine in the regulation of plasma high density and very low density lipoproteins in mice. J Biol Chem 278: 21851–21859. 12668679 10.1074/jbc.M301982200

[4] Zhao Y, Su B, Jacobs RL, Kennedy B, Francis GA, et al (2009) Lack of phosphatidylethanolamine N-methyltransferase alters plasma VLDL phospholipids and attenuates atherosclerosis in mice. Arterioscler Thromb Vasc Biol 29: 1349–1355. 19520976 10.1161/ATVBAHA.109.188672

[5] Jacobs RL, Zhao Y, Koonen DP, Sletten T, Su B, et al (2010) Impaired de novo choline synthesis explains why phosphatidylethanolamine N-methyltransferase-deficient mice are protected from diet-induced obesity. J Biol Chem 285: 22403–22413. 20452975 10.1074/jbc.M110.108514 PMC2903412

[6] Fu S, Yang L, Li P, Hofmann O, Dicker L, et al (2011) Aberrant lipid metabolism disrupts calcium homeostasis causing liver endoplasmic reticulum stress in obesity. Nature 473: 528–531. 21532591 10.1038/nature09968 PMC3102791

[7] Bonaa KH, Njolstad I, Ueland PM, Schirmer H, Tverdal A, et al (2006) Homocysteine lowering and cardiovascular events after acute myocardial infarction. N Engl J Med 354: 1578–1588. 16531614 10.1056/NEJMoa055227

[8] Lonn E, Yusuf S, Arnold MJ, Sheridan P, Pogue J, et al (2006) Homocysteine lowering with folic acid and B vitamins in vascular disease. N Engl J Med 354: 1567–1577. 16531613 10.1056/NEJMoa060900

[9] Menon V, Sarnak MJ, Greene T, Wang X, Pereira AA, et al (2006) Relationship between homocysteine and mortality in chronic kidney disease. Circulation 113: 1572–1577. 16549639 10.1161/CIRCULATIONAHA.105.570127

[10] Friedman AN, Hunsicker LG, Selhub J, Bostom AG, Collaborative Study G (2005) Total plasma homocysteine and arteriosclerotic outcomes in type 2 diabetes with nephropathy. J Am Soc Nephrol 16: 3397–3402. 16162814 10.1681/ASN.2004100846

[11] Yi F, Li PL (2008) Mechanisms of homocysteine-induced glomerular injury and sclerosis. Am J Nephrol 28: 254–264. 17989498 10.1159/000110876 PMC2820346

[12] Cunard R, Sharma K (2011) The endoplasmic reticulum stress response and diabetic kidney disease. Am J Physiol Renal Physiol 300: F1054–1061. 21345978 10.1152/ajprenal.00021.2011 PMC3094049

[13] Lindenmeyer MT, Rastaldi MP, Ikehata M, Neusser MA, Kretzler M, et al (2008) Proteinuria and hyperglycemia induce endoplasmic reticulum stress. J Am Soc Nephrol 19: 2225–2236. 18776125 10.1681/ASN.2007121313 PMC2573014

[14] Bhandary B, Marahatta A, Kim HR, Chae HJ (2012) An involvement of oxidative stress in endoplasmic reticulum stress and its associated diseases. Int J Mol Sci 14: 434–456. 23263672 10.3390/ijms14010434 PMC3565273

[15] Zhang K, Kaufman RJ (2008) From endoplasmic-reticulum stress to the inflammatory response. Nature 454: 455–462. 18650916 10.1038/nature07203 PMC2727659

[16] Ohse T, Inagi R, Tanaka T, Ota T, Miyata T, et al (2006) Albumin induces endoplasmic reticulum stress and apoptosis in renal proximal tubular cells. Kidney Int 70: 1447–1455. 16955111 10.1038/sj.ki.5001704

[17] Zinszner H, Kuroda M, Wang X, Batchvarova N, Lightfoot RT, et al (1998) CHOP is implicated in programmed cell death in response to impaired function of the endoplasmic reticulum. Genes Dev 12: 982–995. 9531536 10.1101/gad.12.7.982 PMC316680

[18] Hartz CS, Nieman KM, Jacobs RL, Vance DE, Schalinske KL (2006) Hepatic phosphatidylethanolamine N-methyltransferase expression is increased in diabetic rats. J Nutr 136: 3005–3009. 17116711 10.1093/jn/136.12.3005

[19] Tachibana H, Ogawa D, Matsushita Y, Bruemmer D, Wada J, et al (2012) Activation of liver X receptor inhibits osteopontin and ameliorates diabetic nephropathy. J Am Soc Nephrol 23: 1835–1846. 23085633 10.1681/ASN.2012010022 PMC3482729

[20] Zou W, Li ZY, Li YL, Ma KL, Tsui ZC (2002) Overexpression of PEMT2 downregulates the PI3K/Akt signaling pathway in rat hepatoma cells. Biochim Biophys Acta 1581: 49–56. 11960751 10.1016/s1388-1981(02)00120-8

[21] Rane MJ, Song Y, Jin S, Barati MT, Wu R, et al (2010) Interplay between Akt and p38 MAPK pathways in the regulation of renal tubular cell apoptosis associated with diabetic nephropathy. Am J Physiol Renal Physiol 298: F49–61. 19726550 10.1152/ajprenal.00032.2009 PMC2806120

[22] Cybulsky AV (2010) Endoplasmic reticulum stress in proteinuric kidney disease. Kidney Int 77: 187–193. 19812538 10.1038/ki.2009.389

[23] Szegezdi E, Logue SE, Gorman AM, Samali A (2006) Mediators of endoplasmic reticulum stress-induced apoptosis. EMBO Rep 7: 880–885. 16953201 10.1038/sj.embor.7400779 PMC1559676

[24] Liu G, Sun Y, Li Z, Song T, Wang H, et al (2008) Apoptosis induced by endoplasmic reticulum stress involved in diabetic kidney disease. Biochem Biophys Res Commun 370: 651–656. 18420027 10.1016/j.bbrc.2008.04.031

[25] Kimura K, Jin H, Ogawa M, Aoe T (2008) Dysfunction of the ER chaperone BiP accelerates the renal tubular injury. Biochem Biophys Res Commun 366: 1048–1053. 18158912 10.1016/j.bbrc.2007.12.098

[26] Dickhout JG, Sood SK, Austin RC (2007) Role of endoplasmic reticulum calcium disequilibria in the mechanism of homocysteine-induced ER stress. Antioxid Redox Signal 9: 1863–1873. 17937580 10.1089/ars.2007.1780

[27] Tu BP, Weissman JS (2004) Oxidative protein folding in eukaryotes: mechanisms and consequences. J Cell Biol 164: 341–346. 14757749 10.1083/jcb.200311055 PMC2172237

[28] Tu BP, Weissman JS (2002) The FAD- and O(2)-dependent reaction cycle of Ero1-mediated oxidative protein folding in the endoplasmic reticulum. Mol Cell 10: 983–994. 12453408 10.1016/s1097-2765(02)00696-2

[29] Zhang Y, Wada J, Hashimoto I, Eguchi J, Yasuhara A, et al (2006) Therapeutic approach for diabetic nephropathy using gene delivery of translocase of inner mitochondrial membrane 44 by reducing mitochondrial superoxide production. J Am Soc Nephrol 17: 1090–1101. 16510762 10.1681/ASN.2005111148

[30] Brezniceanu ML, Lau CJ, Godin N, Chenier I, Duclos A, et al (2010) Reactive oxygen species promote caspase-12 expression and tubular apoptosis in diabetic nephropathy. J Am Soc Nephrol 21: 943–954. 20299359 10.1681/ASN.2009030242 PMC2900966

[31] Wada J, Makino H (2013) Inflammation and the pathogenesis of diabetic nephropathy. Clin Sci (Lond) 124: 139–152. 23075333 10.1042/CS20120198

[32] Velagapudi C, Bhandari BS, Abboud-Werner S, Simone S, Abboud HE, et al (2011) The tuberin/mTOR pathway promotes apoptosis of tubular epithelial cells in diabetes. J Am Soc Nephrol 22: 262–273. 21289215 10.1681/ASN.2010040352 PMC3029899

[33] Inagi R (2010) Endoplasmic reticulum stress as a progression factor for kidney injury. Curr Opin Pharmacol 10: 156–165. 20045381 10.1016/j.coph.2009.11.006

[34] Engin F, Hotamisligil GS (2010) Restoring endoplasmic reticulum function by chemical chaperones: an emerging therapeutic approach for metabolic diseases. Diabetes Obes Metab 12 Suppl 2108–115. 21029307 10.1111/j.1463-1326.2010.01282.x

