# Supplementary figures and images for: Pemt Deficiency Ameliorates Endoplasmic Reticulum Stress in Diabetic Nephropathy
Source: PLoS One. 2014 Mar 25;9(3):e92647. doi: 10.1371/journal.pone.0092647 (PMC3965443; doi:10.1371/journal.pone.0092647)

Figure S1

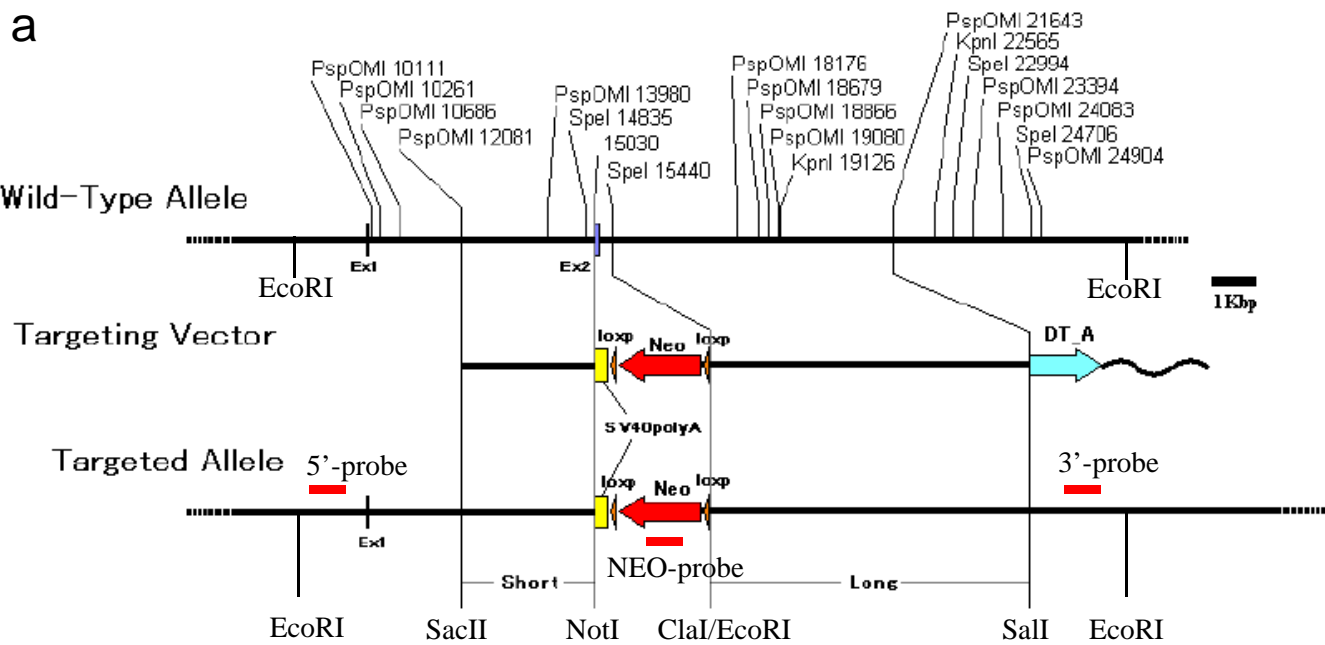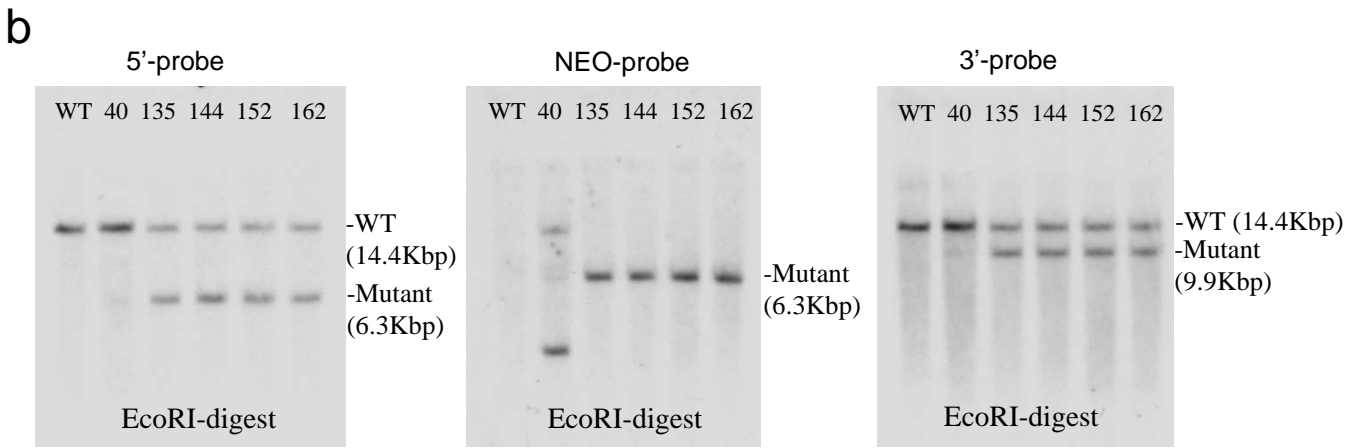

Figure S2

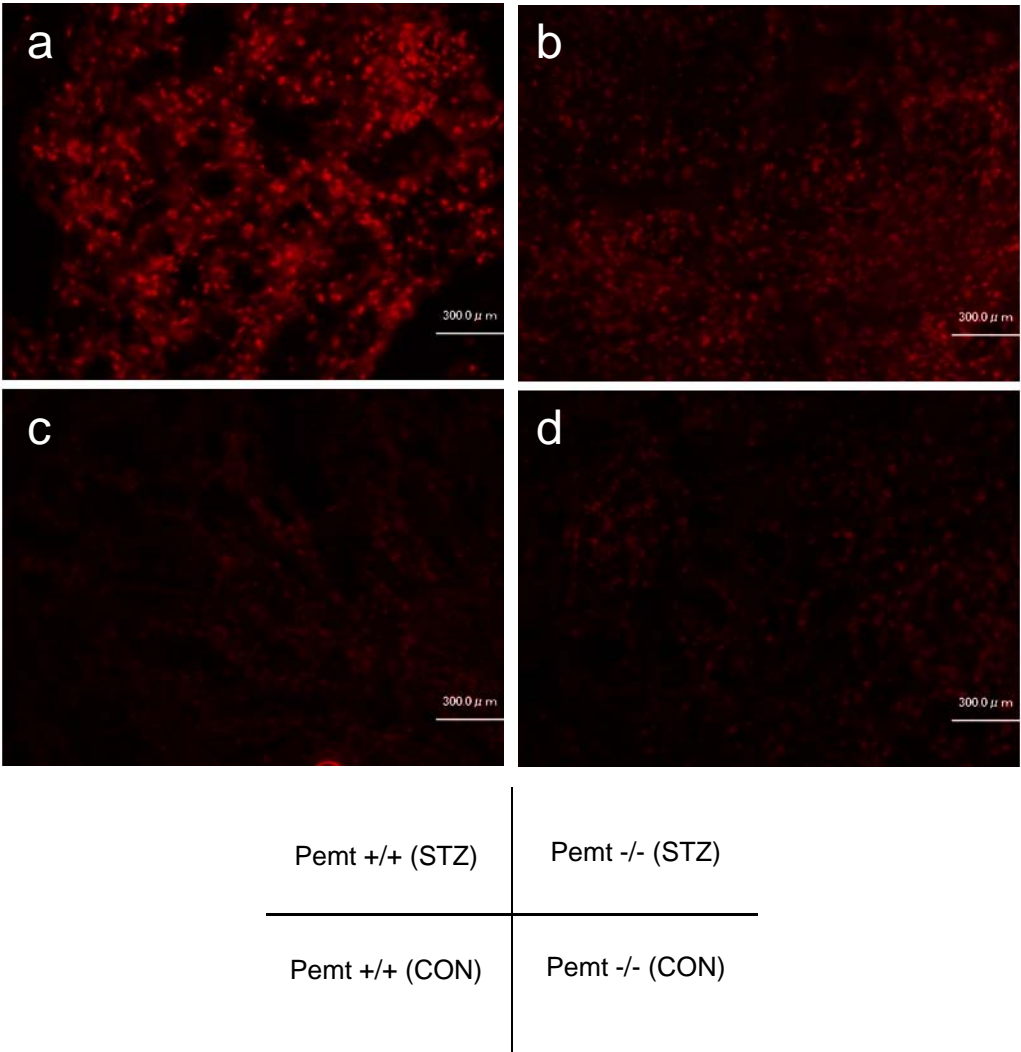

Figure S3

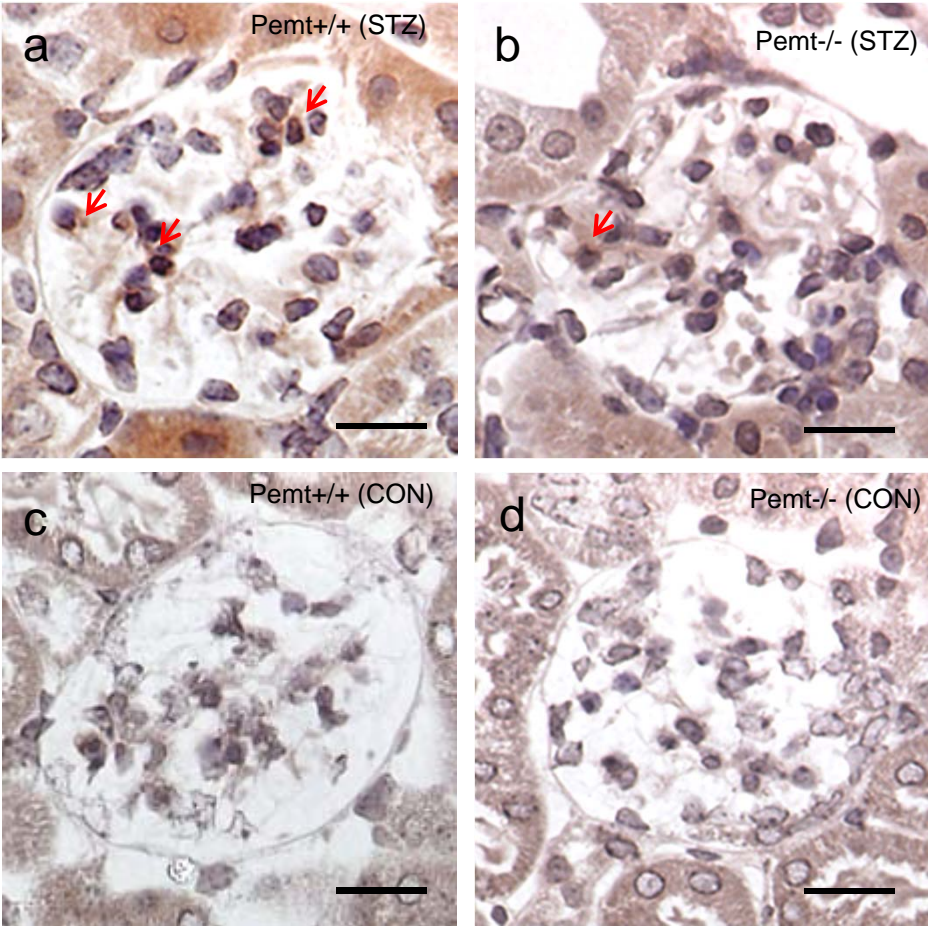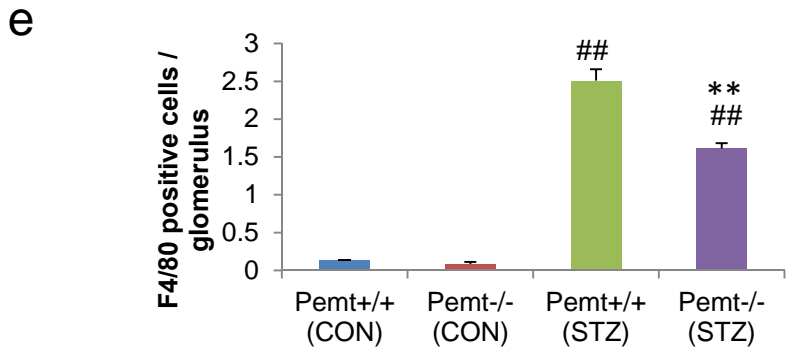

Figure S4

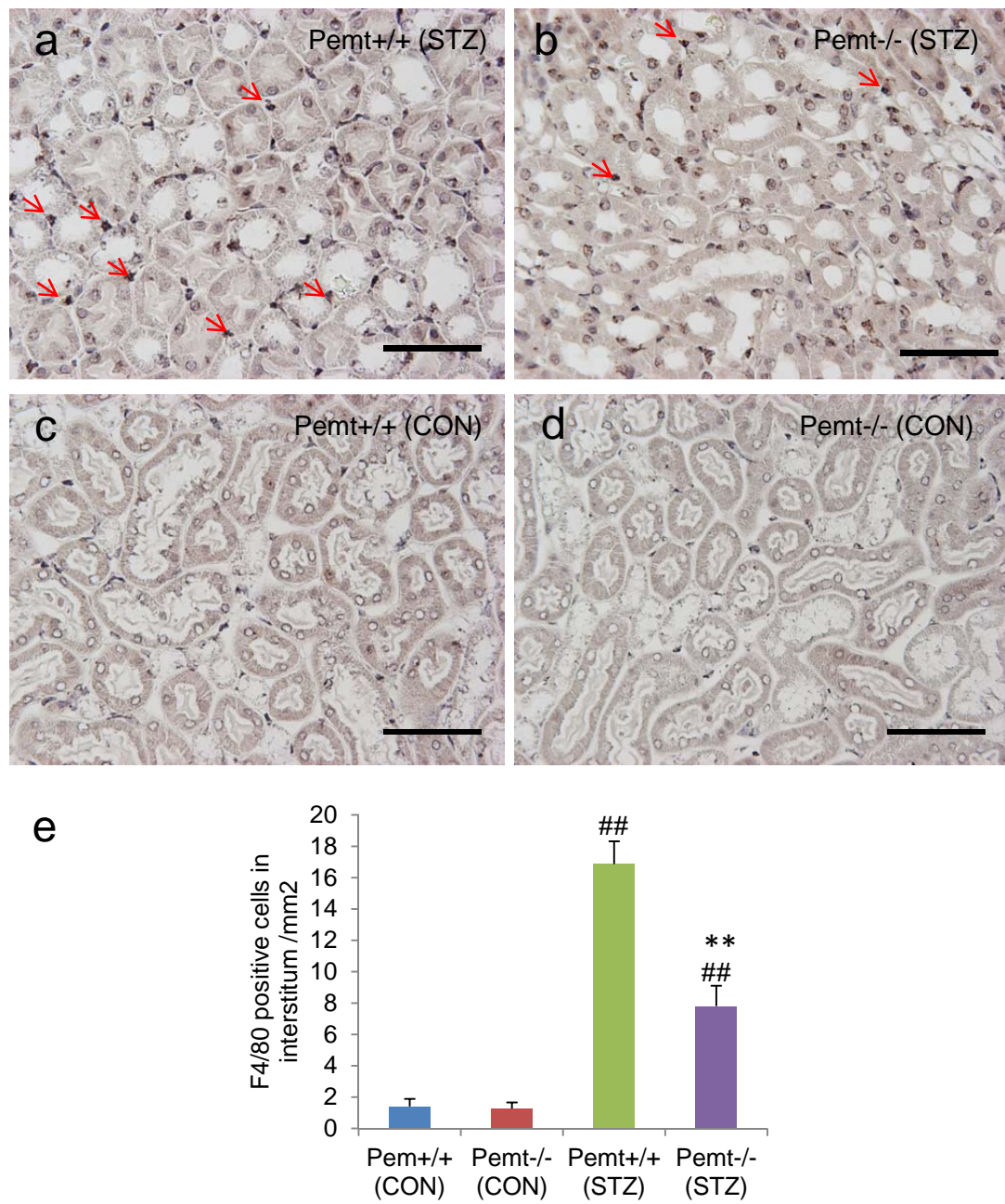

Figure S5

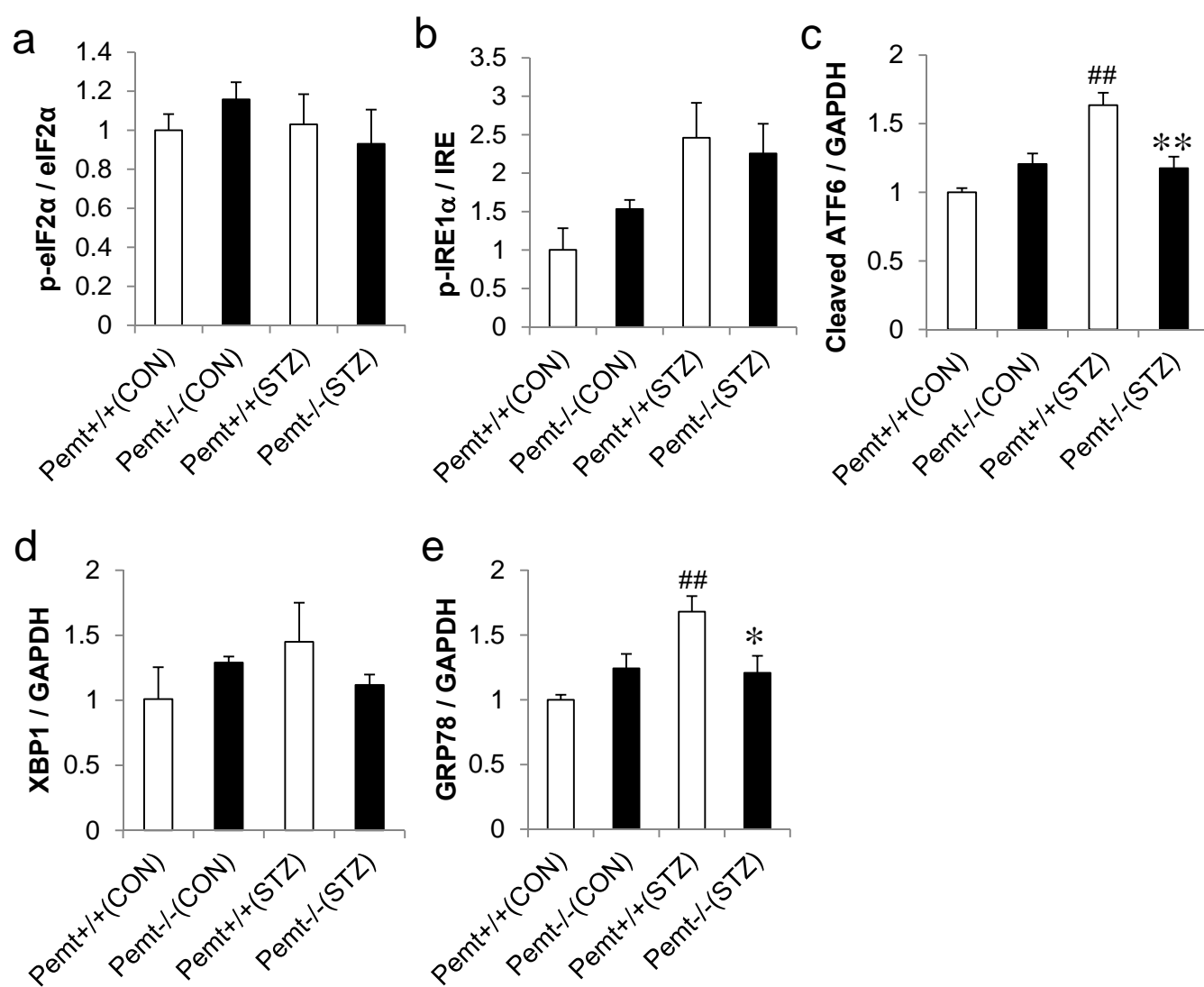

Figure S6

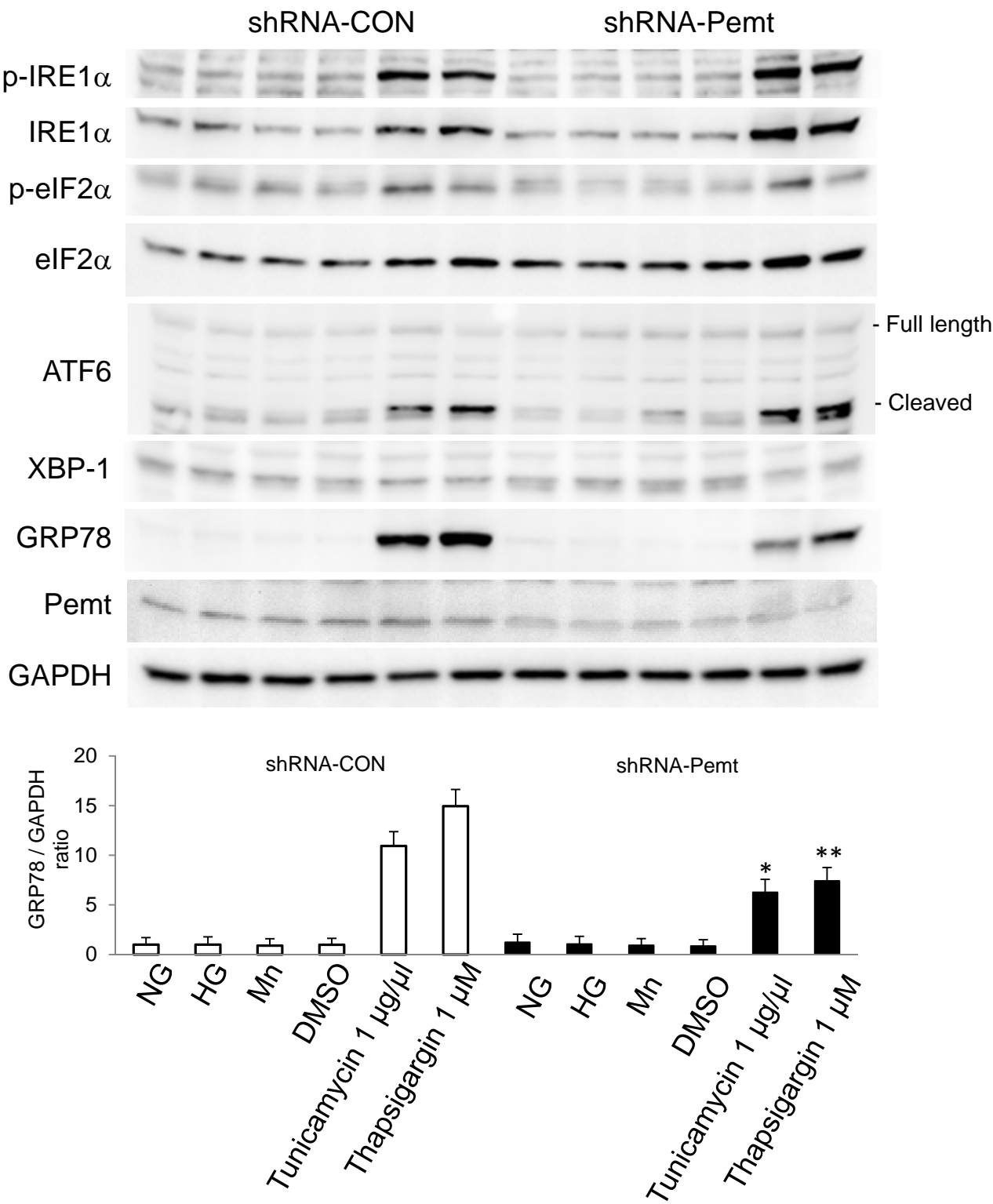

Figure S7

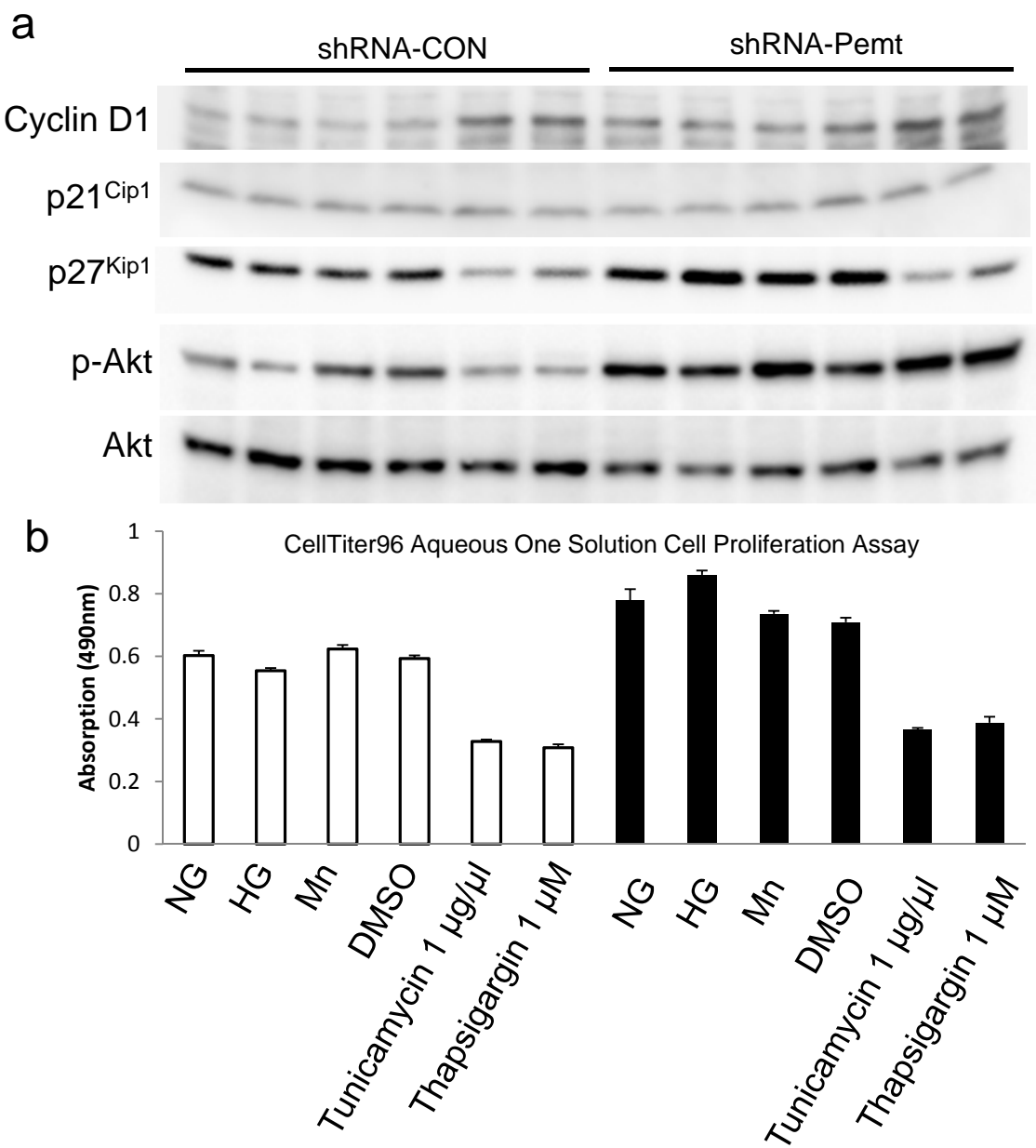

Figure S8

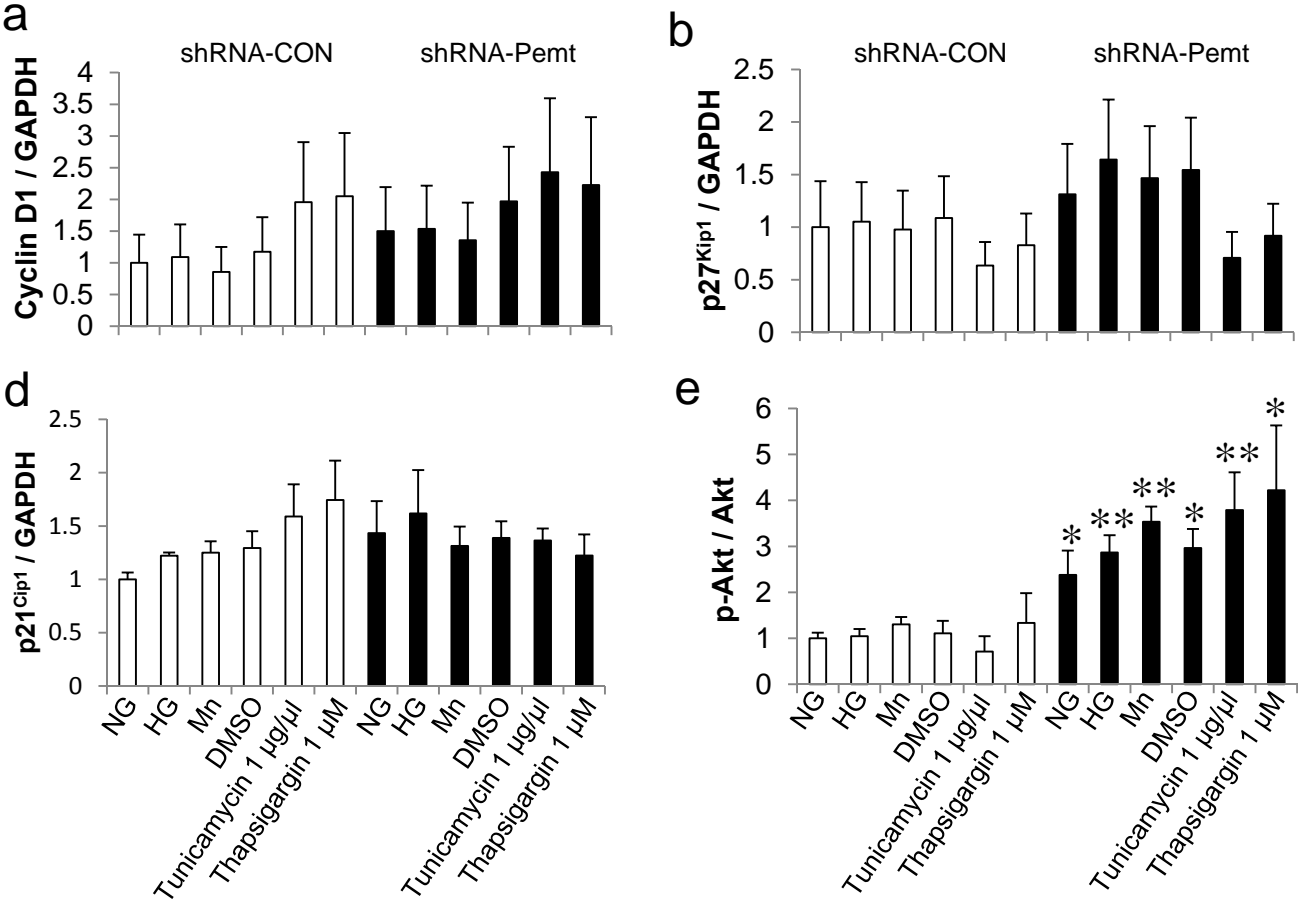

Figure S9

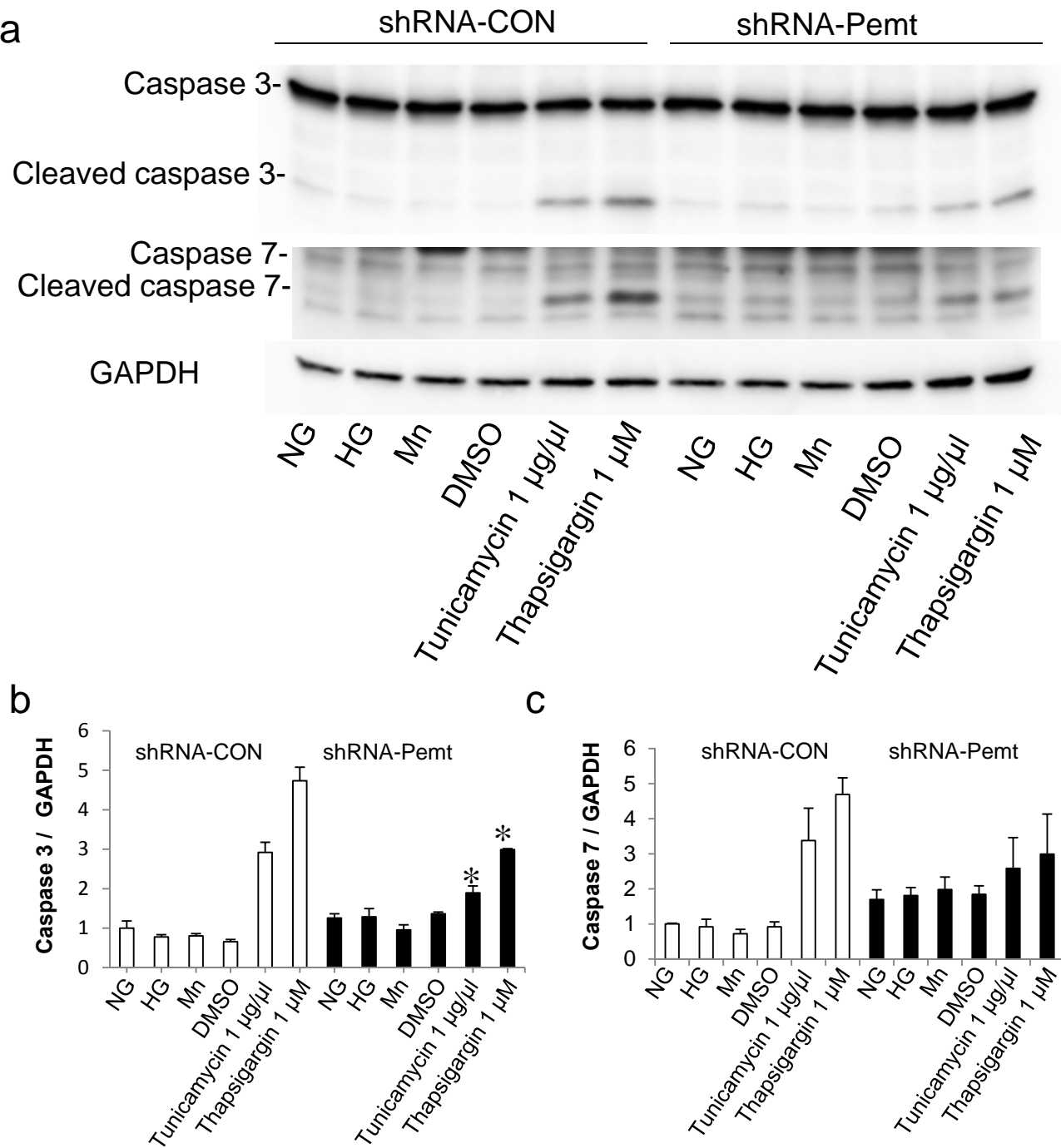

Supplement: File S1 — Figure S1. A schematic diagram of the strategy used to disrupt the mouse Pemt gene. a. Schematic drawings of the Pemt targeting vector. b. The targeted recombination in ES cells (lines 135, 144, 152 and 162) was confirmed by the Southern blot analyses of genomic DNA digested with EcoRI, and the expected sizes of wild-type and Pemt gene-targeted bands using a 5′-probe, 3′-probe and NEO-probe are shown, respectively. Figure S2. Oxidant fluorescence microtopography using hydroethidine in streptozotocin (STZ)-treated diabetic Pemt+/+ and Pemt−/− C57BL/6JJcl mice. The Pemt+/+ and Pemt−/− mice were treated with citrate buffer (CON) or STZ. a–d. The Pemt+/+ (STZ) mice demonstrated a prominent increase in hydroethidine fluorescence, and Pemt deficiency markedly reduced the renal cell-derived superoxide. Bars = 300 μm (a–d). Figure S3. The intraglomerular macrophage infiltration in streptozotocin (STZ)-treated diabetic Pemt+/+ and Pemt−/− C57BL/6JJcl mice. Pemt+/+ and Pemt−/− mice were treated with citrate buffer or STZ. a–d. Immunoperoxidase staining for F4/80, e. The number of F4/80 positive cells/glomerulus. The number of glomerular F4/80-positive cells was significantly reduced in Pemt−/− (STZ) mice compared with Pemt+/+ (STZ) mice. Bars = 20 μm (a–d). ##P<0.01 v.s. Pemt+/+ (CON). **P<0.01 v.s. Pemt+/+ (STZ). Figure S4. The interstitial macrophage infiltration in streptozotocin (STZ)-treated diabetic Pemt+/+ and Pemt−/− C57BL/6JJcl mice. Pemt+/+ and Pemt−/− mice were treated with citrate buffer or STZ. a–d. Immunoperoxidase staining for F4/80, e. The number of F4/80 positive cells in the interstitium per mm2. The number of interstitial F4/80-positive cells was significantly reduced in Pemt−/− (STZ) mice compared with Pemt+/+ (STZ) mice. Bars = 50 μm (a–d). ##P<0.01 v.s. Pemt+/+ (CON). **P<0.01 v.s. Pemt+/+ (STZ). Figure S5. The results of the densitometric analyses of the Western blots of renal cortex tissues from Pemt+/+ and Pemt−/− mice treated with citrate buffer or s [file pone.0092647.s001.pdf]
